# A Scoping Review of Occupational Imbalance Among Korean High School Students: Insights Through the PEO Model

**DOI:** 10.1155/oti/7917526

**Published:** 2026-04-17

**Authors:** Ji-Eun Choi, Yu-jin Jung, Mi-jin Kwon, Myung-Hwa Lee, Soo-jin Park, Sun-Joung Leigh An

**Affiliations:** ^1^ HOPE Parent Training Center, Seoul, Republic of Korea; ^2^ Department of Occupational Therapy, Graduate School of Inje University, Gimhae-si, Republic of Korea, inje.ac.kr; ^3^ The Liberal Arts School, SuSeong University, Daegu, Republic of Korea; ^4^ Department of Multimedia Design, Inje University, Gimhae-si, Republic of Korea, inje.ac.kr; ^5^ Department of Occupational Therapy, Inje University, Gimhae-si, Republic of Korea, inje.ac.kr

**Keywords:** academic stress, Korean high school students, occupational imbalance, PEO model, scoping review

## Abstract

**Background:**

Korean high school students (KHSSs) face immense academic pressures due to the College Scholastic Ability Test (CSAT), a national milestone that determines their future academic and career paths. While extensive societal and governmental support is provided to optimize conditions for examinees, the overwhelming focus on exam preparation disrupts students′ daily routines, leading to significant occupational imbalances that adversely affect their physical, mental, and emotional well‐being.

**Aim:**

This study was aimed at analyzing the factors contributing to occupational imbalance among KHSS using the person–environment–occupation (PEO) model and explore occupational therapy interventions and recommendations to address these challenges.

**Methods:**

A scoping review was conducted, synthesizing research published between 2015 and 2024 in Korean and English. Sixteen studies focusing on KHSS were included, retrieved from databases such as KISS, RISS, DBpia, Web of Science, and PubMed.

**Results:**

Using the PEO model, two key themes were identified. The first, preparation for the CSAT, includes reliance on after‐school private education, nighttime self‐study routines, and unhealthy coping mechanisms such as excessive caffeine use and limited leisure. The second, educational and social climate, reflects the influence of Confucian cultural values that emphasize academic achievement and a hypercompetitive environment fostering peer comparison and anxiety. These factors create a misalignment between KHSS′ personal needs (person), high‐pressure environments (environment), and disrupted daily routines (occupation), resulting in significant physical, emotional, and mental strain.

**Conclusion:**

Addressing these imbalances requires systemic policy reforms to reduce academic pressures, alongside occupational therapy interventions such as stress management programs, lifestyle redesign, advocacy, education, and training programs. Collaboration with psychologists for additional counseling is crucial to support emotional well‐being. These combined strategies can foster resilience, holistic development, and a healthier balance between academic and personal life.

## 1. Introduction

Academic achievement constitutes a primary objective for high school students worldwide, with college entrance examinations representing pivotal milestones in their educational trajectories. In South Korea, this milestone is embodied in the College Scholastic Ability Test (CSAT), a high‐stakes national exam that critically influences students′ academic and career prospects [1]. Unlike in many other countries, where entrance exams are primarily individual undertakings, the CSAT is treated as a societal event, accompanied by substantial governmental and public measures aimed at mitigating stress and facilitating optimal test‐taking conditions [2]. These extensive efforts reflect the cultural emphasis on educational attainment and underscore the pervasive academic pressure experienced by Korean high school students (KHSSs), which extends beyond test day and permeates their daily lives.

To reduce stress and support KHSS on the day of the CSAT, the Korean government implements a variety of nationwide interventions. These include delaying the start of work hours to alleviate traffic congestion, enhancing public transportation services, deploying police officers to assist students in transit, and suspending air traffic during the English listening section to maintain silence in testing environments (Es) [2–4]. While these measures illustrate Korea′s national investment in student success, they simultaneously highlight the systemic intensity of academic expectations, which can interfere with students′ engagement in everyday occupations (Os)—activities essential to health and well‐being.

Against this backdrop, occupational therapy (OT) offers a pertinent framework for understanding how such educational pressures affect students′ daily functioning. OT emphasizes the importance of balanced participation in Os—encompassing self‐care, academic tasks, social interaction, and physical activity—as a foundation for health and emotional resilience [5]. In high‐pressure academic contexts, however, students often sacrifice essential Os, resulting in occupational imbalance, heightened stress, and compromised well‐being [6, 7]. For instance, national survey data reveals that 44% of KHSS regularly skip breakfast, only 14% participate in physical activity, and their stress levels significantly exceed those of peers in other grade levels [8]. These behavioral trends suggest that academic demands may be displacing health‐supportive Os, yet the structural mechanisms underlying these shifts remain insufficiently understood.

**Figure 1:**
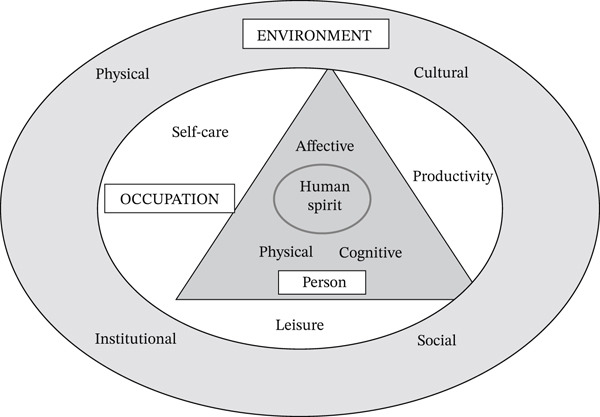
A concept of person–environment–occupation (PEO) model.

Given these concerns, a theoretical framework is required to examine the multifaceted nature of students′ occupational challenges. The person–environment–occupation (PEO) model is particularly well suited for this purpose. This model conceptualizes occupational performance as the dynamic interaction among three interdependent components: the person (P) (e.g., cognitive, emotional, and physical capacities), the E (e.g., social, cultural, and institutional contexts), and the O (e.g., daily activities that are personally meaningful) [9, 10] (see Figure 1). Rather than attributing occupational imbalance to individual deficits, the PEO model highlights how misalignments among these domains can constrain participation and adversely affect mental and physical health.

The versatility of the PEO model has been demonstrated across diverse populations and settings. In Canada, it has guided vocational support interventions for transitioning youth [11]; in Australia, it has improved school participation among adolescents with disabilities by addressing environmental barriers [12]; and in the United States, it has informed inclusive education and aging‐in‐place strategies [13, 14]. These applications attest to the model′s adaptability and cross‐cultural relevance in addressing occupational performance challenges.

In light of the distinctive academic and sociocultural conditions experienced by KHSS, the PEO model offers a robust framework for examining how educational pressures interact with students′ capacities and environmental factors to shape occupational engagement. Despite its potential, the model remains underutilized in Korean educational research, particularly in the context of CSAT preparation. While some domestic studies have examined specific student behaviors—such as oral hygiene or extracurricular participation—they often fail to account for the broader systemic interactions among the P, the E, and the O [15–17].

Therefore, the purpose of this scoping review is to explore how dynamic interactions among personal factors, environmental contexts, and Os contribute to occupational imbalance among KHSS preparing for the CSAT. By applying the PEO model, this review systematically maps and analyzes existing literature to identify patterns of occupational disruption and clarify gaps in research. The findings are intended to inform culturally responsive, evidence‐based strategies for educators, policymakers, and OT practitioners seeking to promote balanced occupational engagement and psychological well‐being among students while also extending the global application of the PEO model to high‐pressure academic contexts.

## 2. Methods

### 2.1. Study Design

This study utilized a scoping review methodology to systematically map the existing literature on occupational imbalance among KHSS preparing for the CSAT. The scoping review design was selected based on its utility in exploring emerging and underexplored topics across heterogeneous sources. The methodological framework was guided by Amaba et al.[18] and Bennett et al.[19] and reported according to the PRISMA‐ScR (Preferred Reporting Items for Systematic Reviews and Meta‐Analyses extension for Scoping Reviews) protocol (2018) to ensure transparency, reproducibility, and methodological rigor [20].

### 2.2. Databases and Search Strategy

A comprehensive search was conducted across both global and Korean academic databases to capture a wide range of relevant literature. Global databases included Web of Science and PubMed, while Korea‐specific databases included Korean Studies Information Service System (KISS), Research Information Sharing Service (RISS), and Database Periodical Information Academic (DBpia). The search was conducted from July 31 to August 8, 2024, using Boolean operators (AND, OR), truncation, and database‐specific filters to maximize relevance. The search strategy was developed based on preliminary exploratory searches using a broad spectrum of keywords related to “adolescents,” “health,” “academic stress,” and “Korea.” This strategy incorporated free‐text searches and included translated synonyms to capture the relevant literature. Furthermore, the keywords were adapted and modified to suit the specific characteristics and indexing systems of each database. The detailed search strategies, including database‐specific search strings, are provided in Appendix B (see Appendix Table B1), while a complete list of the keywords used is presented in Table 1 (see Table 1).

**Table 1 Table A1 Table B1 Table C1 Table D1:** List of keywords utilized for searching.

English keywords	Korean Keywords
KEY 1: [(high school) OR (secondary education) OR (senior high)]	KEY 1: 고등학생 OR 고등학교
KEY 2: [(South Korea) OR (Republic of Korea) OR (Korea)]	KEY 2: 대한민국 OR 한국 OR 국내
KEY 3: [(Academic workload) OR (Extracurricular activities) OR (Education system) OR (Academic culture) OR (Academic performance)]	KEY 3: 학교생활 OR 학업 OR 학교참여
KEY 4: [(Mental health) OR (Physical Health) OR (Physical Activity) OR (Emotional Health) OR (Stress) OR (Wellbeing) OR (Diet) OR (Nutrition) OR (Sleep) OR (Esteem) OR (Alcohol) OR (Smok∗) OR (Academic stress) OR (After‐school activity) OR (Social life) OR (Habits) OR (Routines) OR (Leisure) OR (Self‐care) OR (Quality of Life)]	KEY 4: 건강 OR 정서 OR 스트레스 OR 웰빙 OR 삶의 질 OR 일과

### 2.3. Selection Criteria

To ensure the relevance and consistency of the included literature, this review established explicit selection criteria based on the research aim and scoping review standards. The criteria were designed to include studies that reflect the general experiences of KHSS in relation to occupational imbalance and exclude studies that focused on populations or contexts that do not align with the review′s objectives.

Specifically, inclusion criteria emphasized empirical studies involving KHSS in general educational settings, published between 2015 and 2024 in either English or Korean. Eligible studies addressed themes such as academic stress, lifestyle disruption, health behavior, or participation in daily activities and used quantitative, qualitative, or mixed‐methods designs.

Exclusion criteria were applied to remove studies that focused on specialized student populations (e.g., athletes, students with disabilities and art/military school students), those unrelated to lived school experiences (e.g., curriculum/policy analyses), and those centered outside of South Korea or during the COVID‐19 pandemic. Studies conducted during the COVID‐19 pandemic were excluded to maintain data homogeneity. During this period, in‐person school attendance was largely suspended, and classes were delivered through online platforms (e.g., Zoom or Microsoft Teams), resulting in substantial alterations to students′ usual school routines and occupational patterns. In addition, pandemic‐specific contextual factors, such as social distancing and heightened psychosocial stress, were considered potential confounding influences on occupational imbalance, thereby limiting the comparability and interpretability of findings across studies. These exclusions ensured a consistent population base and cultural context aligned with the review′s scope.

Screening was conducted in two phases by two independent reviewers. Initial screening of titles and abstracts was followed by full‐text assessments. Any disagreements were resolved through team discussion or, when necessary, adjudicated by two independent reviewers. The final criteria are summarized in Table 2.

**Table 2:** Inclusion and exclusion criteria.

Inclusion	Exclusion
1. General Korean high school students	1. Korean elementary school students, middle school students (junior high), and university students
2. Grade 10‐12 (equivalent to high school grades 1‐3 in Korea)	2. Papers targeting foreign high school students
3. Papers in English and Korean	3. Studies addressing challenges or problems experienced by high school students irrespective of country
4. Studies addressing challenges or problems specific to Korean high school culture	4. Tool research, intervention (program) research, and development
5. Qualitative or quantitative research designs	5. Literature reviews
6. General high school students, gifted students, specialized high school students, and autonomous high school students	6. Meister high school and specialized high school students linked to employment after graduation
	7. Alternative school students
	8. Arts and physical education high school students
	9. Military school students
	10. Research focused on education policy, curriculum development, and textbook analysis
	11. High school students with disabilities
	12. Studies that focused on topics related to the COVID‐19 era

### 2.4. Data Gathering and Extraction

The selection of studies was conducted in two distinct phases. Initially, our extensive search across multiple databases yielded 611 articles. After eliminating 37 duplicates, a total of 574 unique records underwent title and abstract screening by three experienced occupational therapists—two native Korean speakers with proficiency in English and one native English speaker. Following a stringent peer review process, the reviewers excluded 492 articles that did not meet the defined inclusion criteria. Subsequently, 82 full‐text articles were retrieved for further evaluation, and following a comprehensive review, 66 articles were excluded due to reasons such as an unsuitable population, methodological discrepancies, or insufficient detail regarding outcomes. Ultimately, 16 studies were retained for final analysis. To guarantee the process′s transparency and replicability, we followed PRISMA guidelines (2020), and a corresponding flow diagram visually represents the study selection procedure.

Following the screening, a detailed data extraction form was designed to systematically collate pivotal study information, including the authors′ names and publication dates, the research methods employed, characteristics of the sample population, major findings and conclusions, and any reported limitations. The three occupational therapists independently carried out the data extraction to ensure uniformity and minimize bias. In order to further verify that the extracted data were closely aligned with the research questions and the field of OT, two additional OT experts reviewed the information. To enhance methodological rigor, the quality of the included studies was appraised narratively during the data extraction process, focusing on methodological characteristics, clarity of reporting, and relevance to the review objectives. A summary of this narrative quality appraisal is provided in Appendix C. (see Appendix Table C1).

**Figure 2:**
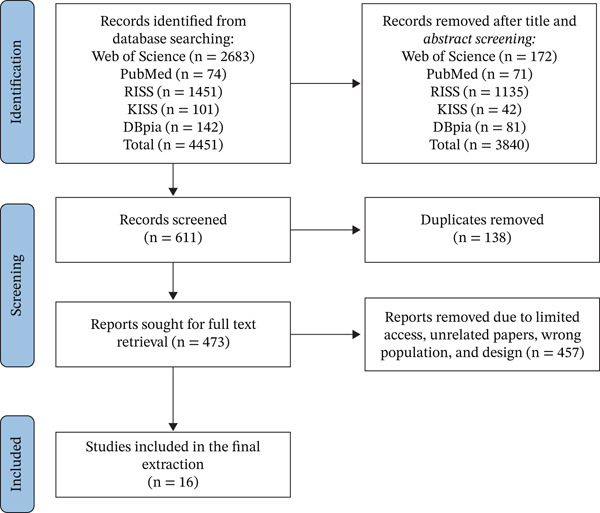
PRISMA flow diagram.

Moreover, ChatGPT‐4 was utilized as an aid in organizing the extracted data, following the methodology described by Alshami et al. [37]. The use of ChatGPT‐4 was strictly limited to language refinement, including grammar and clarity, and did not involve content generation or analytical decision‐making. The authors retained full responsibility for the accuracy and integrity of the extracted data, and all final decisions were made by the authors. Outputs generated with the assistance of ChatGPT‐4 were cross‐checked among the authors and verified against the original study data to minimize potential errors. Finally, all collected data were rigorously cross‐checked for consistency and accuracy prior to finalization. The full review process is visually summarized in the PRISMA‐ScR flow diagram (see Figure 2).

### 2.5. Translation, Paraphrasing, and Transparency Procedures

To ensure transparency and reproducibility, all textual evidence extracted from the included studies—including descriptive findings, participant statements, and author interpretations—was paraphrased rather than directly quoted. Of the 16 included studies, 15 were originally published in Korean and were translated into English using a meaning‐preserving approach to maintain conceptual integrity and alignment with OT terminology. One study was originally published in English; its content was likewise paraphrased rather than directly quoted.

This approach was applied consistently throughout data extraction, analysis, and manuscript preparation. To enhance clarity, accuracy, and interpretive neutrality, translated and paraphrased text was refined through iterative feedback from the supervising professor and researchers with relevant expertise. All interpretive and analytical decisions were made independently by the first author, who retained primary responsibility and oversight of the synthesis process.

To further enhance methodological transparency, Table 3 summarizes the translation, paraphrasing, and validation workflow applied across all included studies. In addition, Appendix D provides a representative bilingual example illustrating the progression from the original Korean source text to the meaning‐preserving English translation and the final paraphrased analytical sentence used in this review (see Appendix Table D1).

**Table 3:** Translation, paraphrasing, and validation workflow.

Step	Workflow stage	Description
1	Text extraction	Relevant textual evidence (descriptive findings, participant statements, and author interpretations) was identified from the included studies.
2	Meaning‐preserving translation	Korean‐language texts were translated into English using a meaning‐preserving approach to ensure conceptual equivalence and alignment with occupational therapy terminology.
3	Paraphrasing	All translated texts were paraphrased, and direct quotations were avoided across all research stages.
4	Expert review and refinement	The paraphrased texts were iteratively refined through feedback from the supervising professor and researchers with relevant expertise.
5	Authorial oversight and integration	Final interpretive and analytical decisions were made independently by the first author, and the procedures were applied consistently across data extraction, analysis, and manuscript writing.

### 2.6. Data Analysis, Coding, and Theme Development

A two‐stage analytical approach was employed to examine and synthesize the selected studies. In the first stage, descriptive statistics were conducted using Microsoft Excel to summarize essential study characteristics, including publication year, research methodology, language, and participant demographics. This step provided a structured overview of the evidence base and informed the direction of the qualitative synthesis.

In the second stage, thematic analysis was conducted using Delve software (2024), applying an inductive and iterative coding process [38]. The three occupational therapists who performed the data extraction independently read and reread the studies to identify meaningful excerpts. Relevant excerpts were initially labeled with open codes such as “daily schedule pressure” and “lack of self‐determination.” These codes were then reviewed iteratively and clustered into subthemes such as “disrupted daily routines due to academic overload”, “deterioration of physical and mental health, “ and “over reliance on private education and erosion of autonomy,” which contributed to broader themes like “csat‐oriented educational culture and its impact on occupational balance.”

In cases where certain codes did not appear consistently across multiple studies, they were initially grouped under provisional categories aligned with the PEO model—such as “occupational challenges” or “environmental constraints.” These preliminary categories served as an organizing framework and were refined throughout the analysis to better reflect the nuances of the data.

Subthemes were only formalized when supported by content drawn from at least four studies, enhancing analytical credibility. During the development of final themes, the research team held weekly consensus meetings to resolve discrepancies and ensure that interpretations were grounded in the data. A peer debriefing process further supported the reliability and cultural sensitivity of the findings. Subsequently, the final themes were systematically organized according to the PEO model, providing a conceptual lens to interpret the patterns of occupational imbalance experienced by KHSSs.

## 3. Results

The scoping review included 16 studies (13 journal articles and 3 dissertations) investigating occupational imbalance among KHSSs. These studies collectively involved 79,707 participants, with gender data reported in 13 studies. Among the reported data, 48.98% were male and 46.17% were female; 4.85% of participants came from studies where gender information was not provided. Most studies employed quantitative methodologies, with cross‐sectional surveys being the most common design (50%). A concise summary of the methodological characteristics, participant profiles, and key findings of each included study is presented in Appendix A for reference (see Appendix Table A1). Percentages are presented solely for descriptive purposes and do not reflect individual‐level outcomes.

### 3.1. Participant Demographics

Table 4 presents the demographic characteristics of participants across the 16 included studies (see Table 4). A total of 79,707 KHSSs participated in the reviewed research. Gender data were reported in 13 studies; within these, 39,040 participants (48.98%) were male and 36,803 (46.17%) were female. Gender data were missing for three studies, which together accounted for 3864 participants (4.85%). All participants were Korean nationals enrolled in general education high schools, with studies primarily focusing on academic stress, lifestyle factors, and school engagement.

**Table 4:** Participant demographics of included studies.

Variable	Details
Total participants	79,707 Korean high school students
Nationality	All participants were Korean
School type	General education high schools
Gender reporting	Reported in 13 studies
	Not reported in three studies (*n* = 3864; 4.85% of total participants)
Gender distribution	Male: 39,040 (48.98%)
	Female: 36,803 (46.17%)
Age information	Not consistently reported across studies

### 3.2. Study Designs

In terms of study design, half of the included studies (*n* = 8, 50%) employed cross‐sectional survey methodologies, followed by correlational studies (*n* = 3, 18.75%) and quasiexperimental designs (*n* = 2, 12.5%). *Q*‐methodology, longitudinal analysis, and exploratory/interpretive approaches were each used in one study (6.25%, respectively). The included studies were published between 2015 and 2023, with the year 2016 exhibiting the highest frequency, accounting for four (*n* = 4, 25%) of the total 16 studies. The overwhelming use of quantitative methodologies (14 of 16 studies) reflects a dominant emphasis on measurable academic and behavioral outcomes (see Table 5). However, the scarcity of qualitative and mixed‐method approaches suggests the need for greater methodological diversity to fully explore the lived experiences and occupational patterns of KHSSs.

**Table 5:** Study designs of included studies.

Category	Details
Total included studies	16 (13 journal articles, 3 dissertations)
Methodological approach	Quantitative: 14 (87.5%)
	Qualitative: 1 (6.25%)
	Mixed‐methods: 1 (6.25%)
Specific study designs	Cross‐sectional: 8 (50%)
	Correlational: 3 (18.75%)
	Quasiexperimental: 2 (12.5%)
	*Q*‐methodology: 1 (6.25%)
	Longitudinal: 1 (6.25%)
	Exploratory/interpretive: 1 (6.25%)
Publication year	2023: 2 (12.50%)
	2022: 1 (6.25%)
	2021: 1 (6.25%)
	2020: 1 (6.25%)
	2019: 2 (12.50%)
	2018: 2 (12.50%)
	2017: 2 (12.50%)
	2016: 4 (25%)
	2015: 1 (6.25%)

### 3.3. Thematic Analysis Results

This scoping review applied the PEO model as an analytical lens to examine patterns of occupational imbalance among KHSS. Two major themes were identified:

1. CSAT‐oriented educational culture and its impact on occupational balance
2. Filial piety in Korea′s Confucian heritage and its influence on occupational imbalance

Each theme includes subthemes that reflect recurring issues across the included studies, with relevant PEO components lightly noted.

#### 3.3.1. Theme 1: CSAT‐Oriented Educational Culture and Its Impact on Occupational Balance

This theme highlights the pervasive influence of Korea′s university entrance examination system, specifically the CSAT, on the occupational lives of KHSS. The CSAT‐centered culture imposes excessive academic demands, distorts students′ daily routines, degrades their health, and fosters an overreliance on private education. The resulting occupational imbalance is characterized by the domination of academic pursuits at the expense of rest, leisure, and self‐regulation.

##### 3.3.1.1. Disrupted Daily Routines Due to Academic Overload.

Across multiple studies, KHSS were found to allocate a disproportionate number of daily hours to academic activities. According to national data, Korean adolescents study approximately 12 h per day—significantly exceeding the OECD average of 6–8 h—and sleep less than 6 h on average, often going to bed after midnight [25, 30, 36]. This imbalance is not only a quantitative excess of study time but also leads to the deterioration of essential Os such as sleep, nutrition, and leisure.

For instance, students often skip meals or rely on snacks due to overlapping schoolwork, night self‐study programs, and private academies [24, 31]. Consistent with the trend of disrupted daily routines, qualitative data revealed students often sacrifice sleep due to extensive homework and consume caffeine to forcibly remain awake and productive [33]. Despite government efforts such as delayed school start policies (e.g., 9 a.m. policy), the total volume of academic work remained unchanged, pushing students into even later night study schedules [35].

During vacation periods, KHSS were reported to condense 3 years of CSAT preparation into a single year of intensive study, with school semesters used for repetitive and advanced learning cycles [21]. Students also commonly attempt to memorize thousands of passages from government‐recommended textbooks such as the EBS series, which forms the basis for the CSAT [21]. This pattern illustrates a profound P–O misfit where essential developmental needs are eclipsed by high‐stakes academic demands.

##### 3.3.1.2. Deterioration of Physical and Mental Health.

The disruption of occupational balance has substantial health consequences for KHSS. Compared to adolescents in other OECD countries, Korean students report lower subjective well‐being and significantly higher levels of depression and suicidal ideation [26, 31]. Academic stress was identified as the leading contributor to this deterioration, often manifesting in symptoms such as headaches, fainting, digestive issues, and anxiety [27, 33]

Notably, female students exhibit higher rates of underweight status due to irregular eating patterns, while male students more frequently present with overweight or obesity due to stress‐related behaviors [31]. This points to a systemic issue in which academic overload, combined with inadequate rest and poor coping strategies, impairs adolescents′ physical health.

The link between educational pressure and self‐injurious behavior has also been documented. Annual increases in teen suicide rates have been partially attributed to the intense competition surrounding university admissions [22]. In this context, academic success is not merely a personal milestone but a sociocultural imperative—further amplifying psychological strain and occupational dysfunction.

##### 3.3.1.3. Over‐Reliance on Private Education and Erosion of Autonomy.

Korean students demonstrate one of the highest rates of participation in private education (*hagwon*) globally, with approximately 73% of high school students enrolled in private classes and spending an average of ₩770,000 (USD 570) monthly [39]. In contrast to other countries where private tutoring supplements formal education, in Korea, it is often perceived as more strategic, personalized, and directly relevant to CSAT preparation [21, 40].

Students increasingly come to regard private education as a default learning method rather than a supplementary resource. One student described relying exclusively on the hagwon curriculum, with little motivation to engage in self‐directed learning or modify study strategies [33].

Moreover, despite the high costs and time investment, students who engage heavily in private education report heightened academic stress, even though their grades are often higher [31]. This paradox indicates that private education, while seemingly beneficial, may further exacerbate the occupational imbalance by crowding out other essential life roles and undermining autonomy.

Taken together, these findings reveal how Korea′s CSAT‐centered educational system generates a complex web of occupational disruptions. The overemphasis on academic achievement—mediated through institutional demands, health‐compromising routines, and privatized learning—ultimately constrains students′ opportunities for balanced engagement across the spectrum of adolescent Os.

#### 3.3.2. Theme 2: Filial Piety (Hyo) in Korea′s Confucian Heritage and Its Influence on Occupational Imbalance

This theme explores how the Confucian value of *Hyo* contributes to occupational imbalance among KHSS. Originally introduced from China, Confucianism has developed uniquely in Korea, with *Hyo*—emphasizing absolute respect and obedience toward parents—occupying a central role in personal ethics and social values [16, 41]. In Korean society, *Hyo* functions not only as a familial virtue but also as a core moral compass shaping one′s life goals and identity.

Unlike Western societies, where parent–child relationships tend to be horizontal and autonomy‐focused, Korean culture′s hierarchical structure—deeply rooted in Hyo—positions academic success as a moral obligation children owe to their parents [40, 42]. Within this cultural logic, KHSS often interpret academic achievement as a way of repaying their parents′ sacrifices, internalizing educational success as a measure of filial duty [16, 41, 43]. This phenomenon leads to a pattern where rest, leisure, and self‐directed Os are deprioritized in favor of academic pursuits, resulting in significant occupational imbalance.

##### 3.3.2.1. Filial Piety and the Vertical Structure of the Parent–Child Relationship.

In the Korean context, parent–child relationships are shaped by more than just biological ties. Grounded in Confucian ideals, parents are viewed as self‐sacrificing figures, while children are expected to reciprocate with social and academic success [16]. Many parents regard their devotion to their children′s education as a moral duty, and children, in turn, feel guilt or shame when failing to meet these expectations [16, 22].

This dynamic has contributed to the emergence of distinctive parenting typologies in Korea that reflect intensive involvement in children′s academic lives. Terms such as *Helicopter moms* (mothers who hover over their children′s academic and career decisions, intervening excessively), *Tiger moms* (mothers who impose high achievement standards and use strict discipline), and *Alpha moms* (mothers who identify and cultivate their children′s talents through strategic planning and information control) illustrate how vertical relational structures manifest in contemporary Korean parenting practices [22].

Cheon [22] and Park [33] have reported that KHSS often prioritize their parents′ wishes over their personal interests, pursuing prestigious university admission as a primary life goal. This dynamic is evident in reports where students feel compelled to pursue high academic achievement primarily to avoid disappointing parents, teachers, and peers, effectively turning academic success into a measure of filial and relational obligation [33].

This relational orientation fosters a hypercompetitive academic E, compelling students to sacrifice essential Os—such as adequate sleep, nutrition, physical activity, and emotional self‐care—in favor of prolonged study [21, 26, 31]. The result is an erosion of occupational balance and heightened physical and psychological exhaustion.

From the perspective of the PEO model, this pattern reveals a pronounced misalignment between the student′s internal resources (P), the cultural and familial expectations (E), and the dominant activity demands of academic work (O). This misalignment is particularly embedded in Korean cultural norms and may not be easily interpreted within individualistic Western frameworks. As such, the influence of Hyo represents a culturally unique driver of occupational imbalance among KHSS, underscoring the need to contextualize adolescent well‐being within the sociocultural fabric of Korean society.

## 4. Discussion

**Figure 3:**
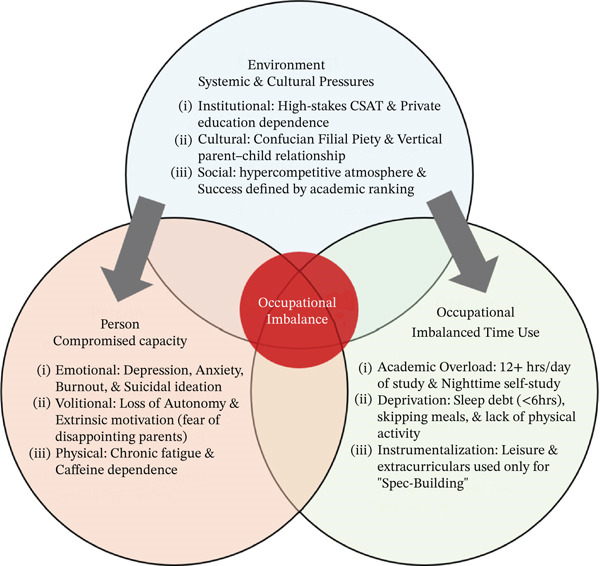
Mechanisms of occupational imbalance among Korean high school students: a PEO perspective.

This scoping review explored the factors contributing to occupational imbalance among KHSS through the lens of the PEO model as a guiding framework. By extending beyond individual‐level academic stress and mental health outcomes, the analysis emphasizes how culturally and institutionally embedded Es interact with personal capacities and daily Os, producing chronic occupational misfit. Previous studies focused primarily on academic stress and mental health outcomes; this review, however, applied the PEO model to examine how mismatches between students′ internal capacities, sociocultural Es, and daily routines lead to broader patterns of occupational misfit, thereby highlighting the systemic and cultural factors contributing to sustained imbalance. To further illustrate the alignment of our findings with the PEO framework, a visual summary of the key results is presented in Figure 3, which maps the identified occupational challenges onto each component of the PEO model. The following sections interpret key findings using each component of the PEO model (see Figure 3).

### 4.1. P: Cultural Internalization, Emotional Burden, and Cognitive Resource Saturation

Understanding the occupational imbalance among KHSS requires an in‐depth examination of personal‐level factors, particularly the influence of cultural values, emotional responses, and cognitive resource allocation on their daily lives and participation in meaningful Os. Within the PEO model, the “P” component encompasses not only physical and cognitive abilities but also motivation, emotional resilience, and identity development—attributes that are deeply embedded within a sociocultural context [9].

In Korea, academic achievement is strongly linked to family expectations and societal norms, which may function as primary motivators for KHSS [16, 22, 42, 44]. Such extrinsic, obligation‐based motivation contrasts with Western contexts, limiting autonomy and reducing self‐directed learning where intrinsic motivation is more emphasized [16, 42, 45, 46]. Many KHSS report studying to meet parental expectations, reflecting internalized duty rather than personal interest [21, 47]. Such cultural expectations may contribute to emotional strain. Academic failure can be internalized as a personal deficiency, increasing guilt, shame, and anxiety, and reducing emotional resilience [22, 23, 33]. National surveys show that 44.3% of high school students report stress, 21.4% depressive symptoms, and 13.5% suicidal ideation, with academic performance as the most prevalent stressor (Korea Youth Risk Behavior Survey, 2023). Prioritization of academic success often leads to disproportionate use of cognitive and emotional resources. For example, an average weekday sleep of 6.2 hours indicates limited recovery and increased risk of occupational imbalance (Korea Disease Control and Prevention Agency, 2023). Excessive academic focus reduces participation in peer, leisure, and community activities, increases interpersonal conflict, and limits physical activity [27–30].

Limited engagement in diverse Os can restrict identity exploration. Overemphasis on academics may hinder resolution of the *identity vs. role confusion* stage, potentially leading to unstable identity formation Dhabhai, 2025; [28, 36]. International comparisons show that Korean students rank high in academic performance but report lower life satisfaction than OECD averages, indicating a persistent misalignment between achievement and well‐being (OECD, 2018). These findings suggest that personal‐level factors, in interaction with environmental and occupational demands, reinforce occupational imbalance among KHSS.

### 4.2. E: Structural Specificities of the Korean Educational Context and Constraints on Occupational Choice

Within the PEO model, the E encompasses physical, social, institutional, and cultural factors that can either facilitate or constrain an individual′s occupational engagement [9]. The occupational imbalance among KHSS arises not only from P factors but from the intersection of sociocultural expectations, high‐stakes academic assessment, institutional practices, and parental involvement, which collectively structure daily Os around narrowly defined academic goals. A primary environmental determinant is the Confucian‐influenced family value system, in which academic achievement is framed as a duty to meet parental expectations rather than a purely personal aspiration [21, 22, 33, 48]. This framing shapes adolescents′ daily activities and prioritization, limiting engagement in other meaningful Os such as leisure, creative activities, or peer interaction [22, 23, 33]. In this sense, cultural expectations operate as environmental pressures that constrain the scope and autonomy of occupational choice [33, 42].

The university entrance examination system constitutes another key environmental constraint. In Korea, preparation for the CSAT dominates the entire high school period, and its high‐stakes nature fosters the perception that public education alone is insufficient [21]. Consequently, private education has become de facto institutionalized. Over 70% of high school students participate in such supplementary education, often extending total study hours to over 10 h per day, which anchors the daily schedule around academic work and limits nonacademic occupational engagement [21, 25, 29, 31, 39].

Parental involvement further reinforces these environmental constraints. Patterns such as the “*helicopter parent*” (excessive oversight), “*tiger parent*” (high achievement under strict control), and “*alpha parent*” (strategic orchestration of talent development) may appear supportive but can undermine adolescents′ self‐determination and occupational autonomy [22, 46]. From a PEO perspective, these social environmental factors exemplify structural constraints on occupational performance, which can indirectly affect identity development and emotional well‐being [9, 46].

Comparative analysis highlights the distinctive intensity of these structural pressures in Korea. While Japan and China share Confucian‐rooted educational aspirations, the prevalence and institutionalization of private supplementary education differ. In Japan, participation in private juku averages around 60% [49, 50]., whereas China′s “Double Reduction Policy” reduces participation to 38%–45% [51, 52]. In contrast, Korean students′ participation remains around 70%, with after‐school schedules densely allocated to supplementary learning, underscoring the rigidity of the educational E [27, 30, 34, 39].

Western educational contexts such as Finland, the Netherlands, and Canada offer a sharp contrast. These systems prioritize holistic development and the quality of learning experiences over purely academic outcomes, and parents typically act as facilitators rather than directive managers, supporting occupational diversity and psychological well‐being [53–55]

Overall, the Korean high school educational E reflects a tightly interwoven system of cultural norms, institutional structures, and parental practices that collectively constrain occupational choice and autonomy. Within the PEO model, this context exemplifies how environmental pressures shape occupational balance, highlighting the importance of culturally informed interpretation and systemic interventions.

### 4.3. O: The Multidimensional Impact of an Academics‐Centered Lifestyle on Occupational Imbalance

Within the PEO framework, O encompasses the meaningful activities individuals engage in daily, which serve as fundamental supports for physical health, emotional stability, identity formation, and overall life satisfaction [9]. However, KHSS exhibit an occupational profile heavily dominated by academic pursuits, resulting in limited diversity and significant imbalance across occupational domains [33]. This imbalance extends beyond time allocation, structurally restricting opportunities for functional engagement and diminishing overall quality of life.

Most KHSS devote over 12 h daily to academic work, including regular classes, self‐directed study, private tutoring, and online lectures [21, 33]. Consequently, essential self‐care Os—such as sleep, nutrition, physical activity, and rest—are consistently compromised, and occupational engagement becomes quantitatively and qualitatively homogenized [26, 27, 31, 33, 34]. Academic tasks are predominantly goal‐oriented and externally regulated, while opportunities for autonomy‐driven, creative, or socially interactive Os steadily decline [30, 33]. In the short term, this pattern leads to physical fatigue and emotional exhaustion; in the long term, it may delay identity development and reduce life satisfaction [22, 29, 33, 46].

Although many KHSS nominally participate in extracurricular activities such as volunteering, clubs, reading, presentations, and self‐directed learning, these activities are often instrumentalized as strategic steps for university admission or resume building [21, 27]. This instrumentalization undermines intrinsic motivation and the inherent value of leisure and interest‐based Os, further restricting autonomy and agency in daily life [22, 46].

O is not merely a means of occupying time; it is a critical pathway for developing functional skills, social roles, and personal identity [5]. However, the structural features of the Korean educational E fail to support balanced participation across diverse occupational domains. Deficits in sleep, physical activity, and regular nutrition lead to functional decline, while restricted opportunities for social engagement and leisure increase emotional exhaustion and social isolation [21, 22, 24], 25]. Alarmingly, many KHSS internalize the perception that nonacademic activities are irrelevant, losing awareness of their occupational choice and autonomy [21, 27].

Cross‐cultural comparisons highlight the structural specificity of the Korean context. Japan and China, sharing Confucian educational traditions, exhibit differing patterns: Japanese students engage more autonomously in extracurricular and club activities with lower reliance on private tutoring, while China′s “Double Reduction” policy seeks to regulate private tutoring and strengthen public schooling, reflecting institutional efforts to restore life balance [49, 51]; Statista, 2023; [52]. In contrast, Korea′s high‐intensity academic culture and institutionalized private education persist throughout the high school years, marginalizing nonacademic Os [21]. Western educational contexts explicitly embed occupational balance as an educational goal. Countries such as Finland, Canada, and Sweden prioritize extracurricular activities—including leisure, volunteering, creative pursuits—as integral to student development and positively weigh them in admissions decisions [23, 56]. According to PISA [57] data, Korean students spend approximately 3 h more daily on academics than the OECD average but rank lowest in sleep and leisure time. This contrast underscores how KHSS are structurally confined to a singular occupational focus, limiting autonomy and participation in diverse meaningful activities.

In conclusion, from a PEO perspective, KHSS experience pronounced occupational imbalance, characterized by limited diversity of meaningful Os, concentration of functional skills in academic tasks, and restricted autonomy and satisfaction in daily activities. This imbalance is primarily shaped by structural and cultural factors rather than individual failings, highlighting unique barriers in the Korean educational context and their relevance for cross‐cultural understanding of adolescent occupational health.

### 4.4. Imbalance in PEO Interactions: Structural Influences on Occupational Performance Among KHSSs

The PEO model posits that human occupational performance results from dynamic and interdependent interactions among three core components: the P, the E, and the O [9]. These components constitute a dynamic system where alterations or constraints in any one domain reverberate across the others, influencing occupational fit and balance [9]. Findings from this study indicate a pronounced disharmony among these components in the occupational engagement of KHSS, manifesting as chronic occupational imbalance.

As individuals, KHSS share universal psychological needs for autonomy, competence, and relatedness and possess intrinsic motivation toward self‐directed occupational participation [46]. Nevertheless, the Korean educational context is characterized by a vertically structured family system, a strong cultural emphasis on filial responsibility, and a high‐stakes, university entrance exam–driven schooling system. This sociocultural and institutional E disproportionately privileges academic achievement while structurally constraining alternative Os, including leisure, self‐care, and social interactions [21, 22, 36, 42].

Extracurricular activities—including volunteering, reading, creative pursuits, clubs, and sports—are often instrumentalized for strategic college admissions purposes, rather than pursued for intrinsic interest or autonomy [21, 23]. As a result, occupational engagement is narrowed, reducing opportunities for meaningful and restorative participation.

This imbalance is further entrenched through feedback loops among the PEO system. Excessive academic demands deplete physical and emotional resources, diminishing occupational performance capacity, which subsequently triggers escalated environmental pressures from parents and schools. These pressures manifest in intensified private tutoring, further reductions in personal time, and constraints on nonacademic engagement, creating a cyclical pattern of resource depletion and occupational restriction that exemplifies a pronounced lack of adaptive occupational fit within the PEO framework [9].

Importantly, this occupational imbalance cannot be reduced to individual stress or motivational deficits. Rather, it reflects structurally embedded and culturally reinforced institutional interactions. Cultural expectations linking academic success to familial obligation, the educational system′s undervaluing of nonacademic Os, and a socioeconomic reliance on private education collectively limit KHSS′ occupational choices and performance [21–23, 36, 42].

Therefore, addressing occupational imbalance requires moving beyond individual‐centered interventions. Restoring balance necessitates multidimensional strategies that coordinate and transform not only individual capacities and autonomy but also environmental structures, institutional policies, and occupational cultures surrounding students. This systemic perspective underscores the importance of considering cultural and institutional contexts when interpreting PEO interactions and designing OT interventions.

### 4.5. Implications for Practice

This review underscores the value of the PEO model as a framework for understanding how students′ internal capacities are strained by rigid Es and narrowly defined occupational roles.

At the practice level, occupational therapists and school psychologists can support students by collaboratively constructing routines that restore balance across occupational domains. This includes intentionally incorporating rest, leisure, and self‐directed activities and guiding students to reflect on the intrinsic vs. extrinsic nature of their academic pursuits. Such interventions strengthen not only self‐regulation but also identity development and volitional engagement.

At the institutional level, educators and policymakers can apply the PEO framework to redesign school systems in ways that promote occupational diversity and psychological well‐being. Policies that encourage flexible scheduling, broadened curricular options, and wellness‐centered benchmarks can help redefine success beyond academic metrics alone. These structural shifts create Es where students have greater autonomy and opportunities to engage in meaningful, varied Os. By applying the PEO model with cultural and developmental sensitivity, practitioners and stakeholders can promote more sustainable and adaptive lifestyles for KHSS—grounded in participation that supports both academic functioning and personal well‐being.

## 5. Limitations and Recommendations

This review provides an OT perspective on the academic and lifestyle imbalance experienced by KHSS. However, several limitations highlight important directions for future research. First, the majority of included studies relied on survey‐based quantitative designs, offering limited insight into how students interpret their academic routines or internalize occupational roles. Future studies should incorporate mixed‐methods approaches to strengthen credibility and deepen context. Combining quantitative trends with qualitative accounts would allow researchers to examine not only what students do but also how they experience and assign meaning to their daily Os—particularly those shaped by social or familial expectations. Second, regional differences in academic pressure were not explored. Future studies should compare how environmental demands, available supports, and cultural expectations vary between urban and rural areas. This would help clarify whether students in rural settings experience less occupational overload and which Es support a better PEO fit. Third, the lack of longitudinal research limits our understanding of long‐term outcomes. Studies that track students into university or adult life could reveal how sustained imbalance affects identity formation, career direction, and well‐being over time. Lastly, this review reflects a broader systems‐level gap: the limited presence of occupational therapists in Korean schools. Expanded OT involvement in educational settings could provide real‐time insights into how academic Es shape student functioning and create opportunities for interventions that support meaningful participation—not just academic performance. Specifically, occupational therapists should recognize the sociocultural influences that shape adolescents′ occupational patterns, design school‐based or community‐level programs to restore occupational balance, and collaborate with educators, parents, and policymakers to promote schedules and learning Es that value rest, play, social engagement, and self‐care as essential for development. Furthermore, culturally tailored strategies, such as parental education programs, could help shift the overemphasis on academic achievement toward more holistic conceptions of well‐being. Additionally, it is important to acknowledge that this review primarily relied on Korean language studies, which may introduce language and methodological dominance bias. Future research should aim for a more inclusive selection process, incorporating international sources and employing a bilingual dual extraction method to ensure a more balanced and globally applicable understanding of KHSS′s occupational health.

## 6. Conclusion

This scoping review offers critical insights into the occupational imbalance experienced by KHSS, analyzed through the lens of the PEO model. The findings reveal that relentless academic demands and Confucian‐rooted societal expectations (E), coupled with exam‐focused routines and self‐directed learning practices (O), create a profound misalignment with KHSS′s individual needs, capacities, and emotional well‐being (P). This imbalance leads to significant physical, mental, and emotional exhaustion, reinforcing a system that prioritizes academic achievement over holistic health and development.

The results highlight the need for systemic reforms that realign educational Es and daily Os with students′ personal needs. The PEO framework underscores the transformative potential of OT in this process—by addressing systemic environmental factors, redesigning Os, and strengthening individual capacities to promote an adaptive PEO fit. Reform begins with advocating for educational policies that embrace diverse definitions of success and ensuring that OT services support KHSS′ mental health and social participation across all levels of the educational system [58, 59].

Occupational therapists are uniquely equipped to design and implement stress management and lifestyle redesign programs [60]. These programs can incorporate evidence‐based approaches such as cognitive behavioral therapy and psychoeducation to enhance key occupational outcomes, including sleep quality and school engagement [60]. Beyond symptom management, these approaches enable KHSS to actively reconstruct their occupational balance and develop self‐regulatory skills for sustained well‐being.

Furthermore, resilience can be fostered through meaningful Os, supported by interventions that align with adolescents′ attributions and leverage their strengths in social and leisure activities [61]. This proactive approach promotes adaptive participation and strengthens students′ sense of belonging, reinforcing the holistic and expanding role of school‐based OT [62].

Effective change requires collaboration among educators, parents, and community stakeholders to ensure balanced schedules and access to mental health services. Culturally tailored interventions, such as parental education, can help shift societal values toward a more holistic understanding of adolescent well‐being. By integrating these multilayered strategies, OT can redefine success in culturally sensitive ways, supporting KHSS in achieving balanced development and providing a model for addressing similar challenges in other high‐pressure educational systems.

These findings extend beyond Korea′s context, reflecting broader educational dynamics across East Asia. In China, the culture of “*score primacy*” driven by high‐stakes exams has been shown to induce systemic stress [63]. In Japan, high rates of student depression highlight the need for targeted emotional skills education [64]. In Singapore, performative, competition‐driven school systems contribute to heightened academic pressure [65]. These parallels suggest that educational cultures emphasizing performance over balance share structural risks to adolescent well‐being, and Korea′s case offers a lens for regional and global reflection.

Considering that the CSAT‐oriented educational culture narrows KHSS′s daily Os to exam preparation while Confucian filial values intensify academic pressure and constrain autonomy, policy reforms should promote diversified evaluation systems, flexible school schedules, and family‐based psychoeducation programs that cultivate supportive and autonomy‐enhancing Es.

## Funding

No funding was received for this manuscript.

## Conflicts of Interest

The authors declare no conflicts of interest.

## Data Availability

The data that support the findings of this study are available from the corresponding author upon reasonable request

## Appendix A

**Table A1:** Summary of the main features and findings of the included studies.

Author (year)	Aim	Sample (male/female)	Document type/research design/language	Results
Jo & Choi (2015) Jo & Choi [21]	The purpose of this study is to investigate the influence of hakwon on high school students. Two research problems were set to define the research direction. First, this study investigated how the contents of hakwon courses in Korea changed as high school students advanced into a higher grade. The researcher found that students chose different hakwon by their grade level and figured there had to be a reason. With this understanding, firstly, this study intended to discover and interpret elements of changes in school curriculum, class, and college entrance examination system in connection to hakwon. Secondly, the meaning of hakwon to Korean high school students was investigated	7	Article exploratory and interpretative analysis Korean	High school students′ experience with hakwon was summarized into three large groups by grade level. First, first‐grade high school students wanted to prepare for internal school exams which are becoming increasingly important and difficult and they wanted to concentrate on math and English, the key subjects in high school education. Secondly, to second grade high school students, hakwon was a place where they can prepare for KSAT and major subjects they are about to learn in earnest. It was meaningful since the administrative dilemma of the high school curriculum triggered by KSAT was resolved. Thirdly, third grade high school students choose EBS KSAT lectures to get high scores in KSAT and this changed the paradigm of KSAT studies. In the meantime, the significance of hakwon education perceived by high school students was classified into four main categories. First, high school students understood hakwon as an institute to provide customized learning to better meet their own situation and necessities. Second, students perceived hakwon as an institute with the greatest influence and responsibility for high school records and college entrance examination, the most important factors in their lives at the moment. Third, high school students considered hakwon as a resting place where they can have conversations about themselves and get practical help. This means they saw hakwon as a place where they could have close relationships with their peer students both in learning and personal life. Fourth, experiences in hakwon tended to undermine students′ own learning abilities. In the long run it seems necessary to ensure education to help students find their goals and grow problem‐solving ability by searching for answers on their own.
Cheon (2016) Cheon [22]	The purpose of this study is to examine stress experience among male high school students across levels of academic achievement using indigenous psychological analysis	355 (355/0)	Dissertation cross‐sectional Korean	First, as for stress experience, a majority of students listed academics, followed by university entrance exam and career. As for emotions in the stressful situation, students listed frustration and anxiety. As for thoughts in the stressful situation, students listed wanting to give up and being worried. As for coping, students listed self‐regulation. Second, as for the person who provided social support in the stressful situation, a majority of students listed friends, followed by parents. As for the type of social support, students listed consoling and encouragement, conversation and counseling and advice. Third, as for differences between levels of academic achievement and interpersonal relationship, there were significant differences in parent–child relationship and relationship with friends, but differences in teacher–student relationship were not found. In other words, students with a high level of academic achievement were more likely to receive emotional support from parents and students with a low level of academic achievement were more likely to experience parental neglect, rejection, and conflict with parents. In addition, students with a low level of academic achievement were more likely to be socially excluded by friends than students with a high level of academic achievement.
Hong (2016) Hong [23]	The purpose of this study is to examine the effect of academic stress on suicidal ideation, and to verify the moderating effect of family cohesion	404 (203/201)	Article correlational Korean	First, academic stress had significance effect on suicidal ideation. The family cohesion has moderating effect on the between academic stress and suicidal ideation. This study explained the implications and limitations of the findings at the practical levels and finally made a number of suggestions for both practices in social welfare.
Kim & Park (2016) Kim & Park [24]	The purpose of this study was to assess the relationship that adolescent stress perception level has with dietary habits and oral health behaviors in high school students	514 (177/337)	Article cross‐sectional survey Korean	The highest stress type was indicated to be academic stress (mean ± standard deviation [SD], 3.09 ± 0.89). The next was shown to be home (family) stress (mean ± SD, 2.85 ± 0.84). The possibility of using a dental clinic was indicated to be less in girls than boys (*p* < 0.001). Regarding subjective oral health behavior, the possibility of visiting a dental clinic was low in those who thought that their own oral health condition was not good or moderate (*p* < 0.05). Also, it was shown that the higher stress led to the higher possibility of visiting a dental clinic (*p* < 0.01). Students with higher grades in the upper ranks were indicated to have a high possibility of having a regular meal (*p* < 0.01). Higher stress led to the significantly higher possibility of eating cariogenic food (*p* < 0.01). Students with median grades had a high possibility of eating cariogenic food (*p* < 0.01), while students with higher grades had a low possibility of eating cariogenic food (*p* < 0.05). These results show that stress perception level influences dietary habits and oral health behaviors. Thus, there is a need to develop a program in high schools to promote the physical and mental health of students to relieve stress. Substantial and systematic oral health education is thought to be likely needed to develop desirable dietary habits.
Seo & Choi (2016) Seo & Choi [25]	The purpose of this study is to examine the difference between sleep duration and the intake of high‐caffeine drinks among high school students using data collected via the Korea Youth Risk Behavior Web‐based Survey Questionnaire, which is a representative survey of Korean youths	35,904 (17,907/17,997)	Article cross‐sectional Korean	Statistically significant differences in sleep duration were found between the group that consumed high caffeine drinks and the group that did not consume high caffeine drinks (*t* = 32.42, *p* < 0.001). There were also statistically significant differences between high caffeine drinks and the relief of fatigue after sleep (*t* = 20.48, *p* < 0.001). In conclusion, we found that the average sleep duration of high school students during the weekdays is very short at 6 h, and that those who consume high‐caffeine drinks sleep less than those who do not. This implies health education and policies on managing proper sleep duration and the intake of high‐caffeine drinks should be provided to improve the mental health of youths.
Choi & Seo (2017) Choi & Seo [26]	This study was conducted to examine the relationship between sleep duration during the weekend and suicidal ideation among Korean high school students	33,336 (17,129/16,207)	Article cross‐sectional survey Korean	Sleep duration during the weekend had a statistically significant impact on suicidal ideation. Those who sleep over six and under 7 h during the weekend were 1.25 times (*p* < 0.001) more likely to have suicidal ideation than those who sleep over 8 h during the weekend. Furthermore, the value was 1.36 times (*p* < 0.001) for those who sleep over 5 and under 6 h, and 1.69 times (*p* < 0.001) for those who sleep under 5 h. We found Korean high school students who sleep under 5 h during the weekend were the most likely to have suicidal ideation. Therefore, high school students need more than 8 h of sleep even on weekends in order to reduce suicidal ideation.
Jeong, Kim & Shin (2017) Jeong, Kim & Shin [27]	The purpose of this study was to examine the moderated mediating effect of maladaptive cognitive emotion regulation strategies through academic stress on the relationship between socially prescribed perfectionism and test anxiety	604 (367/237)	Article correlational Korean	First, using three‐step mediated regression analysis, the significant partial mediating effect of academic stress on the relationship between socially prescribed perfectionism and test anxiety was found. Second, using hierarchical multiple regression analysis, it was found that maladaptive cognitive emotion regulation strategies moderated the relationship between academic stress and test anxiety. Third, using SPSS Macro, the moderated mediating effect of maladaptive cognitive emotion regulation strategies (especially, rumination, self‐blame, and catastrophizing) on the relationship between socially prescribed perfectionism, academic stress, and test anxiety was found.
Ra, Kim & Cho (2018) Ra, Kim & Cho [28]	The purpose of this study was to identify factors associated with intermittent and light smoking among Korean high school students	2851 (2339/512)	Article cross‐sectional survey English	Among all the participants, 1231 (43.2%) were intermittent and light smokers. Factors significantly predicting intermittent and light smoking were gender and grade (biological factors); subjective stress (psychological factor); and mother′s smoking, sibling′s smoking and academic achievement (sociocultural factors). In smoking cessation programs, health care providers both at school and in the community should consider the unique biological, psychological, and sociocultural characteristics of intermittent and light smoking behavior among high school students.
Yun, Kim & Kim (2018) Yun, Kim & Kim [29]	The purpose of this study was to investigate the parallel multiple mediating effects of high school students′ academic stresses between time pressures on subjective well‐being	379 (379/212)	Article cross‐sectional Korean	First, time pressure was correlated with all subvariables of academic stress. Time pressure was negatively correlated with satisfaction in lif, but was positively correlated with negative emotion. On the other hand, time pressure was not correlated with positive emotion. Academic stress was negatively correlated with satisfaction in life but positively correlated with negative emotion. Time pressure was not correlated with positive emotion. Second, Studying and career partially mediate between time pressure and satisfaction of life. Scores and career also partially mediated between time pressure and negative affect.
Bae & Chin (2019) Bae & Chin [30]	The purpose of this study was to analyze the effect of the ordinance, which limits hagwon operating hours to 10 p.m., on the late‐night study time at the hagwon itself and the sleep time of high school students	133 (71/62)	Article quasiexperimental Korean	The average time spent on studying at the hagwon was between 10 p.m. and 12 a.m. and decreased in 2014 in those regions where the revised ordinance has been introduced. There was also no statistically significant difference in the change of sleep time between the regions with the revised ordinance and the other regions. We also discussed why this ordinance has apparently been ineffective on the study time spent by high school students as well as possible ways to increase their sleeping hours.
Ku & Chung (2019) Ku & Chung [31]	The purpose of this study is to explore the transitional relationship between high school students′ latent classes of academic factors and their latent classes of physical health factors	2810	Article Longitudinal analysis Korean	It was found that the “high academic stress, academic achievement, and private education group” had the highest probability of being involved in the ”frequent treatment group” or the “low subjective health satisfaction group.” Based on a person‐centered approach, it was found that the latent group with high risk in terms of their academic factors was likely to belong to a relatively unhealthy latent group. This study confirmed the transitional relationship between academic factors and physical health factors. This implies that it is necessary to select physical health risk groups according to academic factors and to consider effective intervention and tailored approaches which are most appropriate to the subjects.
Lee & Kim (2020) Lee & Kim [32]	This study examined the factors related to skipping breakfast in high school girls	581 (0/581)	Article cross‐sectional survey Korean	The breakfast skipping group consumed more afternoon snacks and late night snacks, and ate dinner irregularly. Although the breakfast skipping group experienced the negative effects of skipping breakfast, they showed low recognition for the importance or role of breakfast.
Park (2021) Park [33]	The purpose of this study was to identify types of general high school students who experience academic burnout, analyze each type characteristics and develop the *Q*‐block for assessment	34 (13/21)	Dissertation *Q*‐methodology Korean	Korean high school students experiencing academic burnout were categorized into five types. Type 1 is “competitive type” that thinks students have to endure academic burnout in order to achieve high academic achievements, but feels negative emotions such as irritation and anger, and feels highly competitive with their peers. Type 2 is the “meaningless type” that has low motivation for school work, because they could not internalize the value or meaning of studying. Type 3 is “functionally deteriorating type” which complains of physical exhaustion, cognitive impairment and psychological distress such as depression and worthlessness. Type 4 is the “fearful type” that perceives one′s past achievements negatively and feels intense anxiety about the upcoming future. Type 5 is “depressed type.” Students categorized as depressed types habitually compare their grades with their friends, feel depressed and lose self‐esteem.
Shin & Choi (2022) Shin & Choi [34]	This study investigates the relationship between sleep duration, sleep quality, time use, and dietary quality of adolescents	423 (112/311)	Article cross‐sectional survey Korean	On weekdays, male students reported getting 6.6 h of sleep, which was significantly higher than the 5.8 h reported by female students. The sleep quality score between male and female students was not significantly different on weekdays and weekends. Comparing the students categorized as getting 6 h of sleep duration on weekdays and 8 h on weekends, a significantly higher total NQ‐A score was obtained for the long sleep duration group of female students on weekdays. In male students who reported increased screen time on weekdays and study time on weekends, there was a greater frequency of short sleep duration. Our data also revealed that the longer the sleep duration higher the NQ‐A score. In addition, higher NQ‐A scores were determined with shorter screen time and more prolonged exercise time.The results suggest that intense study time and excessive use of smartphones have a negative effect on sleep in high school students. In addition, poor sleep quality and lack of sleep are likely to affect eating habits and nutritional status.
Kim & Eom (2023) Kim & Eom [35]	This study examines the effect of the 9 a.m. school start policy on high school students′ learning	1047	Article quasiexperimental Korean	First, the 9 a.m. school start policy did not have a significant impact on students′ class attitudes. Second, the 9 a.m. school start policy significantly increased students′ academic stress. Third, the 9 a.m. school start policy significantly lowered students′ academic achievement in Korean, Mathematics, and English.
Youn (2023) Youn [36]	The purpose of this study is to examine the factors influencing suicidal ideation among adolescents, particularly exploring the impact of academic stress and self‐esteem on suicidal ideation and whether this influence is moderated by social resources	325 (155/170)	Dissertation Correlational Korean	Adolescent academic stress averaged 2.41 (on a 4‐point scale), self‐esteem averaged 3.06 (on a 4‐point scale, indicating above‐average), while social resources such as attachment to friends scored 4.23 (on a 5‐point scale), teacher rapport scored 3.25 (on a 4‐point scale), and parent‐child relationship scored 2.69 (on a 4‐point scale). Suicidal ideation scored 0.26 (on a 7‐point scale).Regarding suicidal ideation based on general characteristics, it was observed that among the individual characteristics of high school students, those in the second grade and those reporting poor subjective health had higher levels of suicidal ideation. Academic stress influenced suicidal ideation, and while this influence decreased when controlling for self‐esteem, it remained significant, indicating that academic stress affects suicidal ideation partly through self‐esteem mediation. It was found that the higher the academic stress, which is a cause of stress, the higher the suicidal ideation, the higher the self‐esteem, the lower the suicidal thoughts, and the better the parental relationship, which is a social resource, the lower the suicidal ideation. Attachment to friends and teacher rapport showed no statistically significant correlation with suicidal ideation. Interaction effects between academic stress and parent–child relationships significantly influenced suicidal ideation. Ultimately, perceived academic stress and self‐esteem among Korean high school students were Seoidentified as factors influencing suicidal ideation, while the parent–child relationship was predicted as a regulating factor for suicidal ideation.

## Appendix B

**Table B1:** Search strategies.

Database	Search strings	Limits/filters	Date searched
Web of Science	(Field 1) (high school) OR (secondary education) OR (senior high)(Field 2) (South Korea) OR (Republic of Korea) OR (Korea)(Field 3) (Mental health) OR (Physical Health) OR (Physical Activity) OR (Emotional Health) OR (Stress) OR (Wellbeing) OR (Diet) OR (Nutrition) OR (Sleep) OR (Esteem) OR (Alcohol) OR (Smok∗) OR (Academic stress) OR (Academic workload) OR (Extracurricular activities) OR (Education system) OR (Academic culture) OR (Academic performance) OR (After‐school activity) OR (Social life) OR (Habits) OR (Routines) OR (Leisure) OR (Self‐care) OR (Quality of Life)	Year: 10 years (2015~)	July 31, 2024
PubMed	((high school)) AND ((South Korea) OR (Republic of Korea) OR (Korea)) AND ((Mental health) OR (Physical Health) OR (Physical Activity) OR (Emotional Health) OR (Stress) OR (Wellbeing) OR (Diet) OR (Nutrition) OR (Sleep) OR (Esteem) OR (Alcohol) OR (Smok∗) OR (Academic stress) OR (Academic workload) OR (Extracurricular activities) OR (Education system) OR (Academic culture) OR (Academic performance) OR (After‐school activity) OR (Social life) OR (Habits) OR (Routines) OR (Leisure) OR (Self‐care) OR (Quality of Life))	Year: 10 years (2015~)	July 31, 2024
RISS	((대한민국)OR(한국)OR(국내))AND((고등학생)OR(고등학교))AND((학교생활)OR(학업)OR(학교참여))AND((건강)OR(정서)OR(스트레스)OR(웰빙)OR(삶의 질)OR(일과))	Year: 10 years (2015~)	August 2, 2024
KISS	((대한민국)OR(한국)OR(국내))AND((고등학생)OR(고등학교))AND((학교생활)OR(학업)OR(학교참여))AND((건강)OR(정서)OR(스트레스)OR(웰빙)OR(삶의 질)OR(일과))	Year: 10 years (2015~)	August 1, 2024
DBpia	((대한민국)OR(한국)OR(국내))AND((고등학생)OR(고등학교))AND((학교생활)OR(학업)OR(학교참여))AND((건강)OR(정서)OR(스트레스)OR(웰빙)OR(삶의 질)OR(일과))	Year: 10 years (2015~)	August 2, 2024

## Appendix C

**Table C1:** Study quality overview of the included studies.

Author (year)	Study design	Aim clearly stated	Sampling described	Data collection clearly described	Analysis clearly described	Overall quality notes
Jo & Choi (2015) [21]	Qualitative	Yes	Yes	Yes	Yes	Exploratory and interpretative analysis with a focused sample; gender composition not specified
Cheon (2016) [22]	Quantitative	Yes	Yes	Yes	Yes	Cross‐sectional dissertation‐based study with a male‐only sample
Hong (2016) [23]	Quantitative	Yes	Yes	Yes	Yes	Correlational study design
Kim & Park (2016) [24]	Quantitative	Yes	Yes	Yes	Yes	Cross‐sectional survey study design
Seo & Choi (2016) [25]	Quantitative	Yes	Yes	Yes	Yes	Cross‐sectional study design
Choi & Seo (2017) [26]	Quantitative	Yes	Yes	Yes	Yes	Cross‐sectional study design
Jeong, Kim & Shin (2017) [27]	Quantitative	Yes	Yes	Yes	Yes	Correlational study design
Ra, Kim & Cho (2018) [28]	Quantitative	Yes	Yes	Yes	Yes	Cross‐sectional survey study design published in English
Yun, Kim & Kim (2018) [29]	Quantitative	Yes	Yes	Yes	Yes	Cross‐sectional study design
Bae & Chin (2019) [30]	Quantitative	Yes	Yes	Yes	Yes	Quasiexperimental study design
Ku & Chung (2019) [31]	Quantitative	Yes	Yes	Yes	Yes	Longitudinal analysis with limited reporting of sample gender composition
Lee & Kim (2020) [32]	Quantitative	Yes	Yes	Yes	Yes	Cross‐sectional survey study design conducted in a girls′ high school
Park (2021) [33]	Mixed	Yes	Yes	Yes	Yes	Dissertation‐based study using *Q*‐methodology with a focused sample
Shin & Choi (2022) [34]	Quantitative	Yes	Yes	Yes	Yes	Cross‐sectional survey study design
Kim & Eom (2023) [35]	Quantitative	Yes	Yes	Yes	Yes	Quasiexperimental study design; sample gender composition not specified
Youn (2023) [36]	Quantitative	Yes	Yes	Yes	Yes	Dissertation‐based correlational study

## Appendix D

**Table D1:** Representative bilingual example of translation and paraphrasing.

Korean source text	Initial English translation	Final paraphrased English text	Validation note
저는 스스로 공부 방법이나 방식을 바꾸지는 않고 학원에서 알려준 대로 그대로 해요. 힘들어도 그냥 가르쳐준 대로 … 학원 방식대로 해요.	I do not change my study methods or approaches on my own; I simply follow what the hagwon teaches me. Even when it is difficult, I just continue studying in the way I was instructed, following the hagwon′s approach.	One student described relying exclusively on the hagwon curriculum, with little motivation to engage in self‐directed learning or modify study strategies.	Meaning preserved; terminology aligned
